# Long Cycle Life Organic Polysulfide Catholyte for Rechargeable Lithium Batteries

**DOI:** 10.1002/advs.201902646

**Published:** 2019-12-23

**Authors:** Dan‐Yang Wang, Yubing Si, Wei Guo, Yongzhu Fu

**Affiliations:** ^1^ College of Chemistry Zhengzhou University Zhengzhou 450001 P. R. China

**Keywords:** dipyridyl polysulfide, lithium batteries, molecular dynamic simulations, organosulfide, ultra performance liquid chromatographyquadrupole time‐of‐flight‐mass spectrometry (UPLC‐QTof‐MS)

## Abstract

Organic compounds with active sites for lithiation can be used as electrode materials for lithium batteries. Their tunable structures allow a variety of materials to be made and investigated. Herein, a spectrum of dipyridyl polysulfides (Py_2_S*_x_*, 3 ≤ *x* ≤ 8) is prepared in electrolyte by a one‐pot synthesis method from dipyridyl disulfide (Py_2_S_2_) and elemental sulfur. It renders up to seven dipyridyl polysulfides (i.e., Py_2_S_3_, Py_2_S_4_, Py_2_S_5_, Py_2_S_6_, Py_2_S_7_, and Py_2_S_8_) which show fully reversible electrochemical behavior in lithium batteries. In the discharge, the initial lithiation occurs at 2.45 V leading to the breakage of S_α_—S_β_ bonds in Py_2_S*_x_* and formation of lithium 2‐pyridinethiolate, in which lithium is coordinated in between N and S atoms. The left sulfur species act as elemental sulfur, showing two voltage plateaus at 2.3 and 2.1 V. The molecular dynamics simulations show the attraction between pyridyl groups and lithium polysulfides/sulfide via N···Li···S bonds, which enable good retention of soluble discharge products within electrodes and stable cycling performance. In the recharge, low‐order Py_2_S*_x_* (e.g., Py_2_S_3_, Py_2_S_4_, and Py_2_S_5_) remain as the charged products. The mixture catholyte exhibits superlong cycle life at 1C rate with 1200 cycles and 70.5% capacity retention.

## Graphical abstract

A spectrum of Py_2_S*_x_* (i.e., Py_2_S_3_, Py_2_S_4_, Py_2_S_5_, Py_2_S_6_, Py_2_S_7_, and Py_2_S_8_) is prepared in electrolyte by a one‐pot synthesis method, which exhibits superlong cycle life of 1200 cycles with 70.5% capacity retention. Its redox reaction mechanism in lithium batteries is revealed.

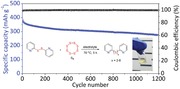

With the vigorous development of portable electronic devices and electric vehicles in the world, the demand for high‐energy storage devices is increasing dramatically.^1^ Accordingly, rechargeable lithium batteries have received tremendous attention.^2^ However, current lithium‐ion (Li‐ion) batteries have been constrained by their inherent capacity limitations (<250 mAh g^−1^) and metal resources.^3^ Therefore, other types of electrode materials with higher capacities and higher energy densities are being explored.^4^ Sulfur is an abundant element on Earth, having a high theoretical specific capacity of 1672 mAh g^−1^.^5^ Each sulfur atom can take up to 2 Li^+^ and 2 e^−^ in the discharge of lithium–sulfur (Li–S) batteries, forming lithium sulfide (Li_2_S).^6^ Recently, organopolysulfides containing a chain of sulfur as active lithiation sites have shown unique electrochemical behavior in lithium batteries.^7^ In addition, they have advantages of abundant resources, high capacities, and tunable structures.^8^

The S—S bonds in organopolysulfides break and reform upon lithiation and delithiation in lithium batteries, respectively.^9^ In 1988, Visco and DeJonghe studied organodisulfides as cathode materials in batteries.^10^ In recent years, some new organopolysulfide molecules and polymers with long sulfur chains have been developed and investigated in rechargeable lithium batteries. For example, dimethyl trisulfide,^11^ diphenyl trisulfide,^12^ and tetrasulfides^13^ have been proven to be promising cathode materials. Trithiocyanuric acid with three nitrogen atoms was used to prepare three‐dimensionally interconnected sulfur‐rich polymers.^14^ Recent studies on organopolysulfide compounds containing N‐heterocycles have shown unique properties and performance. For instance, thiuram polysulfides show stable cycling performance, which is partially due to the adsorption of lithium sulfide/polysulfide by the N^+^ center of the heterocycles in the discharged product.^15^ In addition, N‐heterocycles like pyridine in dipyridyl disulfide (Py_2_S_2_) can enable high discharge voltage plateau at 2.45 V.^16^ In light of the aforementioned, it is clear to see that the dipyridyl polysulfides (Py_2_S*_x_*, 2 < *x*) possessing high theoretical specific capacities are worth investigation and could be a class of promising cathode materials.

Herein, Py_2_S_2_ and one equivalent sulfur were reacted in the Li–S electrolyte at 70 °C for 5 h. The target compound is dipyridyl trisulfide (Py_2_S_3_) with a theoretical specific capacity of 425.4 mAh g^−1^, each Py_2_S_3_ molecule could take 4 Li^+^ and 4 e^−^ when electrochemically reduced in lithium batteries. A mixture of dipyridyl polysulfides (Py_2_S*_x_*) dissolved in the electrolyte was obtained (**Scheme** 1). To confirm the composition of the product, ultra performance liquid chromatography‐quadrupole time‐of‐flight‐mass spectrometry (UPLC‐QTof‐MS) was employed. In the total ion chromatogram (TIC) of the product, Py_2_S*_x_* containing different amounts of sulfur are shown. Among them, A represents unreacted Py_2_S_2_. From B to G, Py_2_S_3_, dipyridyl tetrasulfide (Py_2_S_4_), dipyridyl pentasulfide (Py_2_S_5_), dipyridyl hexasulfide (Py_2_S_6_), dipyridyl heptasulfide (Py_2_S_7_), and dipyridyl octasulfide (Py_2_S_8_) are presented, respectively, indicating the maximum *x* for a stable dipyridyl polysulfide is 8. The e peak is attributed to the electrolyte. The corresponding *m*/*z* values are shown in Figures S1 and S2 in the Supporting Information. In addition, Table S1 in the Supporting Information depicts the proportion of their contents in the ionization mode. Except the unreacted Py_2_S_2_, the yield of polysulfides reaches ≈78%. The obtained catholyte was added in carbon nanotube (CNT) paper to be used as cathode.^17^ Scanning electron microscopy (SEM) and energy‐dispersive X‐ray spectroscopy (EDS) elemental mapping images of the surface of the prepared electrode shown in Figure S3 in the Supporting Information reveal the network of porous CNT paper and even distribution of sulfur and nitrogen elements of Py_2_S*_x_* within the electrode.

**Scheme 1:**
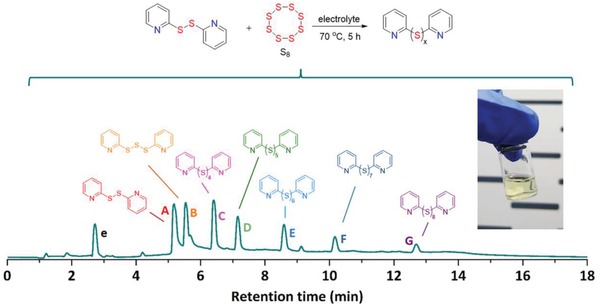
The synthesis of a spectrum of Py_2_S*_x_* in electrolyte and TIC spectrum of the mixture catholyte; the inset is a photograph of the prepared Py_2_S*_x_* catholyte.

The electronic structure is vital factor affecting the electrochemical behavior of organic electrodes.^18^ With the elongation of the sulfur chain of Py_2_S_2_ to that of Py_2_S_8_ (**Figure** 1a), the lowest unoccupied molecular orbital (LUMO) energies show a gradient decrease from −0.06 eV (Py_2_S_2_) to −1.09 eV (Py_2_S_7_), although Py_2_S_8_ displays a bit higher energy level of −0.97 eV. Based on frontier molecular orbital theory, it is generally considered that the electrons are first injected into the LUMO orbitals during the lithiation process, thus making the high order Py_2_S*_x_* with low LUMO energies (e.g., Py_2_S_6,_ Py_2_S_7_, and Py_2_S_8_) lithiated first in the discharge of lithium batteries.

**Figure 1:**
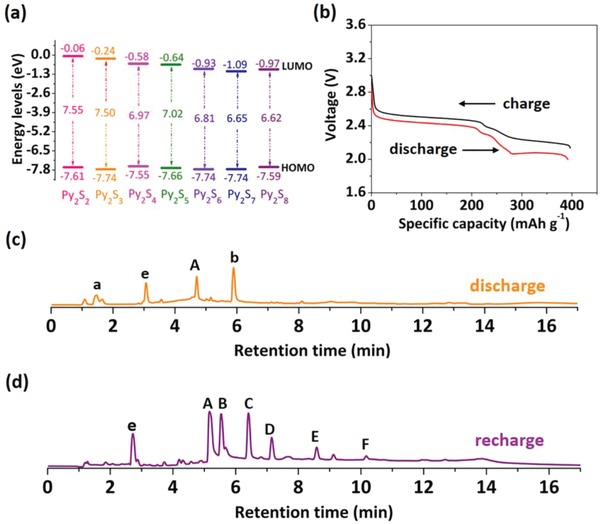
a) Energy levels of the seven Py_2_S*_x_*; b) the first discharge and recharge voltage curves at C/10 rate; c) TIC of the Py_2_S*_x_* electrode after discharge; d) TIC of the Py_2_S*_x_* electrode after recharge.

The electrochemical behavior of Py_2_S*_x_* in batteries is evaluated in lithium half cells, which were discharged and charged galvanostatically. The initial discharge and recharge voltage profiles at C/10 rate are shown in Figure 1b. They consist of three voltage regions. In the discharge, it shows a long discharge plateau at 2.45 V which is due to the formation of lithium 2‐pyridinethiolate.^16^ The following two voltage plateaus at 2.3 and 2.1 V are characteristic of sulfur reduction to form lithium polysulfides and lithium sulfide, respectively. The recharge has similar voltage plateaus with little overpotential. The cell exhibits an initial discharge capacity of 391.7 mAh g^−1^, which is about 92.1% of its theoretical capacity. The high discharge voltage plateau occupies almost 50% of the capacity, which is consistent with the 2 e^−^ transfer to form two lithium 2‐pyridinethiolates per molecule. If the average molecular structure is considered as Py_2_S_3_, the middle sulfur atom takes the other 2 e^−^ to form one lithium sulfide (Li_2_S). The corresponding cycling performance of this cell is shown in Figure S4 in the Supporting Information. Over 100 cycles, the capacity is attenuated to 314.6 mAh g^−1^ which is 80% of the initial discharge capacity. The Coulombic efficiency is over 99.5%.

The cycled products were examined by UPLC‐QTof‐MS. The TIC of the discharged product (Figure 1c) shows peak a, the corresponding MS indicates it is protonated 2‐pyridinethiol, i.e., a form of 2‐pyridinethiolate in MS. Interestingly, peak b implies a product of Py_2_S_2_ with two lithium atoms. This may be due to the formation of a simple coordination structure of N···Li···S in the discharged product, in which the overall charge is neutral and the S—S bond may not break. A trace amount of unreacted Py_2_S_2_ A still exists. Figure S5 in the Supporting Information gives the corresponding *m*/*z* values of three discharge products a, b, and A. The TIC of the recharged product shows the reformation of various Py_2_S*_x_* (Figure 1d; Figure S6, Supporting Information). However, Py_2_S_8_ cannot be regenerated and other Py_2_S*_x_* have been reduced considerably. The proportion of these polysulfides is also shown in Table S1 in the Supporting Information. After 100 cycles, the recharged products range from Py_2_S_2_ to Py_2_S_5_ (Figure S7, Supporting Information) and the other high‐order Py_2_S*_x_* has depleted. Meanwhile, in the Fourier transform infrared (FTIR) spectra (Figure S8, Supporting Information), the stretching band of S–S linkage generally appears in the range of 520–400 cm^−1^. Py_2_S_2_ shows two bands at 426 and 428 cm^−1^ and Py_2_S*_x_* shows weak stretching band in this range. After discharge, almost no band of S–S linkage can be seen. Upon recharging, the reappearance of the band at 424 cm^−1^ illustrates the reformation of S—S bonds. The FTIR results are consistent with those of UPLC‐QTof‐MS.

To further reveal the discharged products of Py_2_S*_x_*, the GFN‐xTB Hamiltonian was used to generate different conformers and rotamers,^19^ the 20 ps trajectory molecular dynamics (MD) simulations with 792 atoms for PySLi and Li as well as Li_2_S was performed with the semiempirical density functional theory (DFT) method, which includes the D3 dispersion correction accounting for the London dispersion energy.^20^ As shown in **Figure** 2a, the total energies of the discharge complexes decrease dramatically at the first 200 fs and begin to converge from 500 fs. Driven by the weak electrostatic interaction, the N atoms of pyridyl groups dynamically form the N···Li bonds network with Li atoms nearby, and provide abundant active sites to embrace the Li and Li_2_S.^16^ The electrostatic potential of the lithiated (discharge) and delithiated (recharge) processes are analyzed in Figure 2b. In Py_2_S*_x_*, the blue surface shown in the electrostatic potential (ESP) energy maps is the most favorable region for electrophilic attack, which mainly arises near N and S atoms. After lithiation, Li atom primarily localizes between N and S sites and forms the stable state of “like complexes.” Indeed, the electron localization function (ELF) analysis further provides a clear signification of that the electron density around N and S atoms shrinks slightly due to the animation of Li atom. Considering there is no valence bond formed, the weak interaction between Li and N/S is easily interrupted by the external electronic field. Thus, at the moment of delithiation, the unperturbed electron density distribution is regenerated, and then the radicals of PyS• reform and are ready for the next cycle of charge.

**Figure 2:**
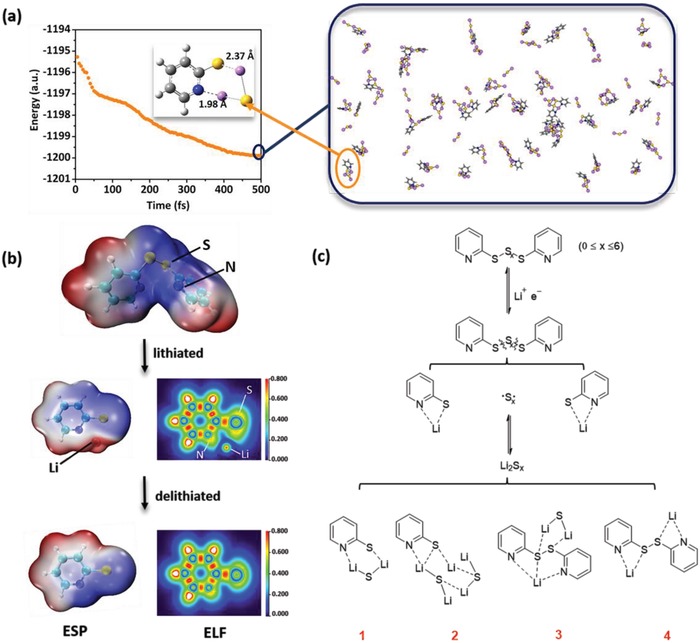
a) The molecular dynamics simulations with 792 atoms for PySLi and Li_2_S; b) the electrostatic potential and electron localization function analysis of Py_2_S*_x_* in the lithiation and delithiation processes; c) the illustration of proposed lithiation process in batteries, complexes **1**–**4** are either simulated or detected by UPLC‐QTof‐MS. The dotted and solid lines represent non‐covalent interactions and covalent bonds, respectively.

From the discharge curve (Figure 1b) and above analysis, the redox processes of Py_2_S*_x_* can be illustrated in Figure 2c. Lithium ions first attack both α‐S in Py_2_S*_x_*, followed by the S_α_—S_β_ bond cleavage. Due to the strong electrostatic attraction by the N atoms of the pyridyl groups, weak coordination bonds between N and Li are formed (i.e., the linkage of N···Li···S). The *m*/*z* value of the protonated 2‐pyridinethiol is obtained through the UPLC‐QTof‐MS analysis of the discharge product (Figure 1c). The sulfur chain in the middle of Py_2_S*_x_* may stay in the state of radicals or convert to stable octasulfur rings, which are converted to lithium polysulfides and finally lithium sulfide. Interestingly, the 2‐pyridinethiolate that formed N···Li···S cyclic complexes **1**–**3** were also conjectured from DFT calculations (Figure 2a; Figure S9, Supporting Information). Around **2** and **3**, more pyridyl groups may also be coordinated by Li. Meanwhile, the complex **4** is confirmed from UPLC‐QTof‐MS result of the discharged sample (Figure 1c). Compounds **3** and **4** are two unexpected discharged products with Li atoms coordinated but S—S bonds untouched in the structure. A dimer of PySLi is also possible to be compound **4**. Accordingly, these demonstrate that the N atoms on pyridyl groups have strong bonding effects on lithium and lithium polysulfide, thus promoting cycle stability.

Subsequently, the cycling performance of the Li/Py_2_S*_x_* cell at 1C rate are shown in **Figure** 3a. In the superlong‐term cycling, the cell delivers an initial capacity of 388.4 mAh g^−1^ and end capacity of 273.8 mAh g^−1^ after 1200 cycles retaining 70.5% capacity retention. The Coulombic efficiency of all the cycles exceeds 99.5% except the first 15 cycles. Figure 3b shows selected discharge/charge voltage profiles, which reveal the main discharge plateau of 2.35 V followed by a small step plateau. The cutoff voltage at 2.0 V avoids the formation of Li_2_S at 1C rate. Along with the cycle progress, there is no obvious overpotential increase even after 1200 cycles. Notably the cell still has excellent performance when tested at a high rate of 5C, as shown in Figure 3c. It can deliver an initial capacity of 273.7 mAh g^−1^ and retain 62.5% of the initial capacity after 1000 cycles. The Coulombic efficiency exceeds 99.5% in almost all cycles, indicating that Py_2_S*_x_* have highly reversible charge–discharge behavior at high C rates. The selected voltage profiles are shown in Figure S10 in the Supporting Information. Furthermore, a Li/Py_2_S*_x_* cell with a high mass loading of 5.3 mg cm^−2^ was also examined. At C/2 rate, the cell shows a high areal capacity of 2.2 mAh cm^−2^. It still shows stable cycling performance with a first discharge capacity of 220 mAh g^−1^ and capacity retention of 89% over 200 cycles (Figure S11, Supporting Information).

**Figure 3:**
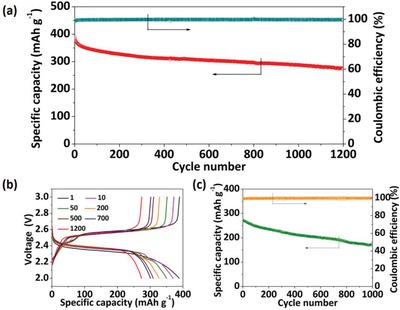
a) Long‐term cycling performance of Li/ Py_2_S*_x_* cell at 1C rate; b) selected voltage–capacity profiles of the cell; c) long‐term cycling performance of a Li/Py_2_S*_x_* cell at 5C rate.

In summary, a spectrum of Py_2_S*_x_* in Li–S electrolyte has been synthesized from Py_2_S_2_ and sulfur as a catholyte for lithium batteries. UPLC‐QTof‐MS analysis confirms the variety of discharged and recharged products. The lithium cell provides a favorable environment for the electrochemical synthesis of Py_2_S*_x_* in the charge process. Molecular dynamics simulations and UPLC‐QTof‐MS results reveal the dynamic network coordinated by the N···Li···S bonds in the discharged products. The mixture catholyte exhibits 1200 cycles with 70.5% capacity retention at 1C rate. This study reveals the intriguing redox reactions of Py_2_S*_x_* and provides guidance for the development of high‐capacity and long‐cycle‐life organic cathode materials for lithium batteries.

## Conflict of Interest

The authors declare no conflict of interest.

## Supporting information

Supporting Information
