# Supplementary material for: Long Cycle Life Organic Polysulfide Catholyte for Rechargeable Lithium Batteries
Source: Adv Sci (Weinh). 2019 Dec 23;7(4):1902646. doi: 10.1002/advs.201902646 (PMC7029628; doi:10.1002/advs.201902646)
Supplement: Supplementary file 1 — Supporting Information [file ADVS-7-1902646-s001.pdf]

Copyright WILEY-VCH Verlag GmbH & Co. KGaA, 69469 Weinheim, Germany, 2019.

ADVANCED SCIENCE
Open Access

Supporting Information

for *Adv. Sci.,* DOI: 10.1002/advs.201902646

Long Cycle Life Organic Polysulfide Catholyte for Rechargeable Lithium Batteries

*Dan-Yang Wang, Yubing Si, Wei Guo, and Yongzhu Fu**

Supporting Information

Long Cycle Life Organic Polysulfide Catholyte for Rechargeable Lithium Batteries

Dan-Yang Wang,[†] Yubing Si,[†] Wei Guo, and Yongzhu Fu*

D.-Y. Wang, Prof. Y. Si, Prof. W. Guo, Prof. Y. Fu
College of Chemistry
Zhengzhou University
Zhengzhou 450001, P. R. China

* E-mail: yfu@zzu.edu.cn (Y. Fu)

[†] These authors contribute equivalently

Experimental Section:

Materials

Commercial lithium-sulfur electrolyte (1.0 M lithium bis(trifluoromethanesulfonyl)imide and 0.15 M lithium nitrate in dimethoxyethane/1,3-dioxolane), and 2,2'-dipyridyl disulfide ($C_5H_4NSSC_5H_4N$, 98%), sulfur powder, chromatographic acetonitrile (LC/MS, Fisher), were purchased and used as received.

Preparation of Dipyridyl Polysulfide Catholyte

2,2'-Dipyridyl disulfide (0.25 mmol, 55 mg) and sulfur powder (0.25 mmol, 8 mg) were mixed in 1 mL of Li-S electrolyte in an Argon-filled glove box. The mixture was stirred and heated at 70 °C for 5 h. The concentration of the dipyridyl polysulfide (Py_2S_x) catholyte is 0.25 M. The high concentration catholyte of 1.2 M was prepared from 2,2'-dipyridyl disulfide (0.6 mmol, 132 mg) and sulfur powder (0.6 mmol, 19.2 mg) in 500 μL of electrolyte.

Preparation of Carbon Nanotube Current Collector

160 mg of carbon nanotubes (CNTs, Nanostructure and Amorphous Materials, Inc.) were dispersed in a miscible solution of de-ionized water (500 mL) and isopropyl alcohol (20 mL) by ultra-sonication for 15 min, followed by vacuum filtration to render a free-standing CNT paper. The CNT paper was dried in an air-oven for 24 h at 100 °C before being peeled off and punched out into circular disks with 1.2 cm diameter (1.13 cm^2, 3-4 mg). The thickness of the CNT paper is about 170-175 μm.

Characterizations

Ultra Performance Liquid Chromatography-Quadrupole Time-of-flight-Mass Spectrometry (UPLC-QTof-MS) was performed on Waters Acquity Plus and Xevo G2-XS QTof equipment. The solvent phase for the LC is from 20/80% to 80/20% acetonitrile (ACN)/water (H_2O) between 0-4 minutes, then it is 80/20% ACN/H_2O between 4-17 minutes. The discharged and charged cathodes were immersed in 1 mL chromatographic ACN separately and the solution was fully shaken by hand. 10 μL of this solution was taken with a pipette and added to a vial with 1 mL of additional chromatographic ACN. The same gradient elution method was used for all the samples. The injection volume was 1 μL and the solvent phase velocity was 0.2 mL min^{-1}. Then these samples were tested at the electrospray ionization (ESI) positive mode. The chromatographic column is Acquity UPLC BEH C18 with 2.1 × 100 mm, 1.7 μm particle size (Waters). Scanning electron microscopy (SEM) of the electrodes were performed on a Zeiss Sigma 500 SEM apparatus. The elemental mapping was examined with energy–dispersive X–ray spectroscopy (EDS) attached to the SEM. X-ray diffraction (XRD) data were collected on a Rigaku Miniflex 600 instrument equipped with Cu Kα radiation. The samples were protected by Kapton tape. The scanning rate is 0.5° min^{-1}, and 2θ is between 20° and 80°. Fourier transform infrared (FTIR) spectra were recorded on a NEXUS 470 infrared spectrometer. Scans in the range of 400-4000 cm^{-1} were recorded.

Cell Fabrication and Electrochemical Evaluation

Coin cells CR2032 were fabricated in the glove box. First, 20 μL of Py_2S_x catholyte was added into the CNT paper current collector. A Celgard 2400 separator was placed on the top of the CNT paper electrode followed by adding 20 μL of electrolyte on the top of the separator. Subsequently, lithium metal anode was placed on the separator. The cell was crimped and taken out of the glove box for testing. This cell configuration has been demonstrated to be effective in evaluating soluble active material in lithium batteries. Cyclic

voltammetry (CV) was performed on a BioLogic VSP potentiostat. The potential was swept from open circuit voltage to 2.05 V and then swept back to 3.0 V at a scanning rate of 0.05 mV s^{-1}. All cells were galvanostatically cycled at 2.0-3.0 V on a LAND battery cycler at different C rates (1C = 425.4 mA g^{-1}, based on the mass of Py_2S_x in the cells).

Computational Details

Computations were carried out with the Gaussian 09 program package.[1] The atom statistics of each molecule were performed with Multiwfn package.[2] Energies and geometries of the intermediates of Py_2S_x ($2 \leq x \leq 8$) were calculated at the M06-2X/cc-pVTZ level, with the 'ultrafine' grid was used. The optimized stationary points were further characterized by harmonic vibrational frequency analysis to ensure that real local minima were reached. The solvent effect is mimicked by using the solvation model based on density (SMD), and the static dielectric constant of dimethoxyethane was set to $\epsilon = 7.07$.[3]

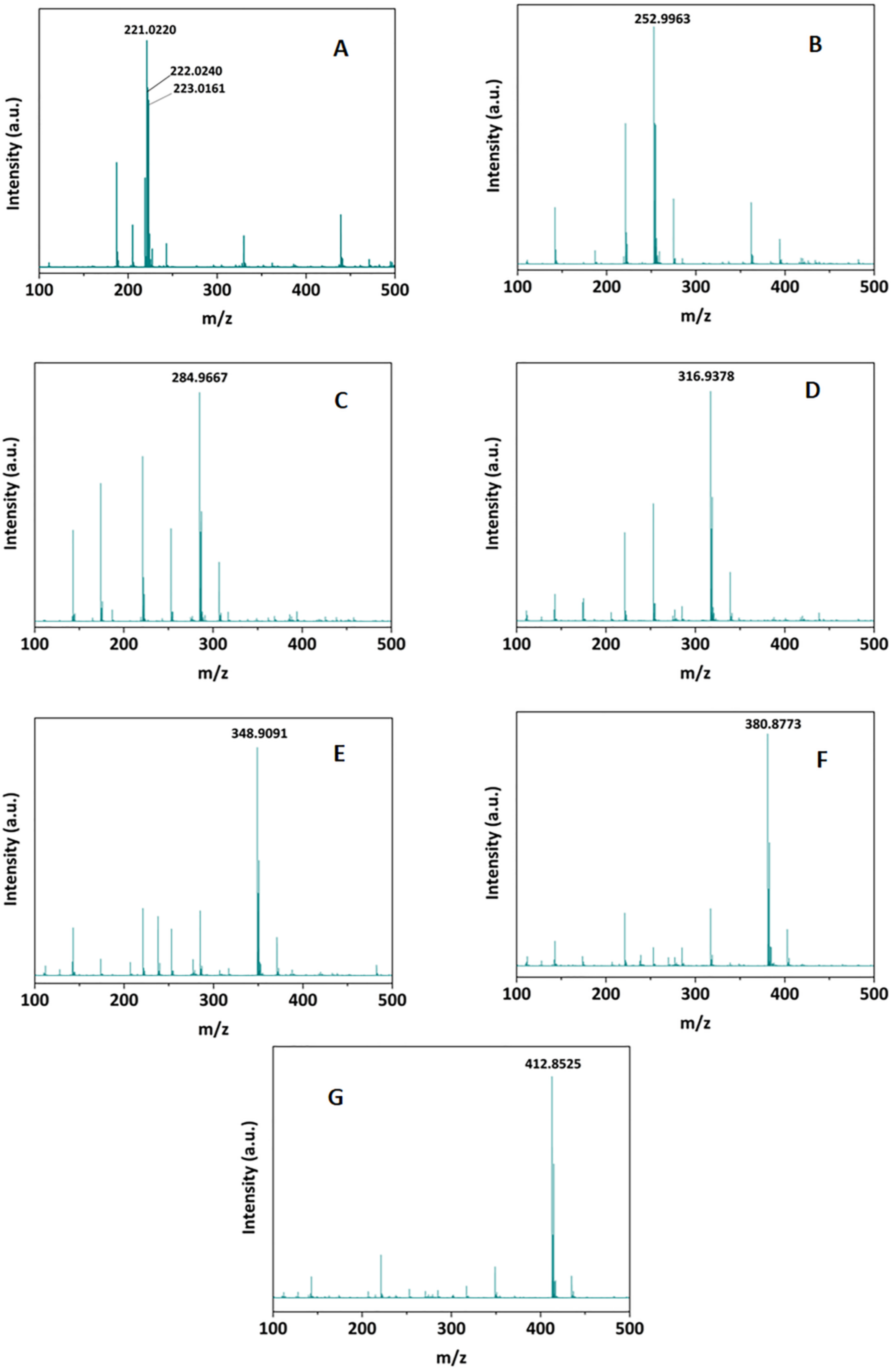
221.0220
222.0240
223.0161
A
Intensity (a.u.)
m/z
252.9963
B
284.9667
C
316.9378
D
348.9091
E
380.8773
F
412.8525
G
100
200
300
400
500

Figure S1. The mass spectra of peaks shown in the UPLC-QTof-MS spectrum (Scheme 1) of the prepared catholyte.

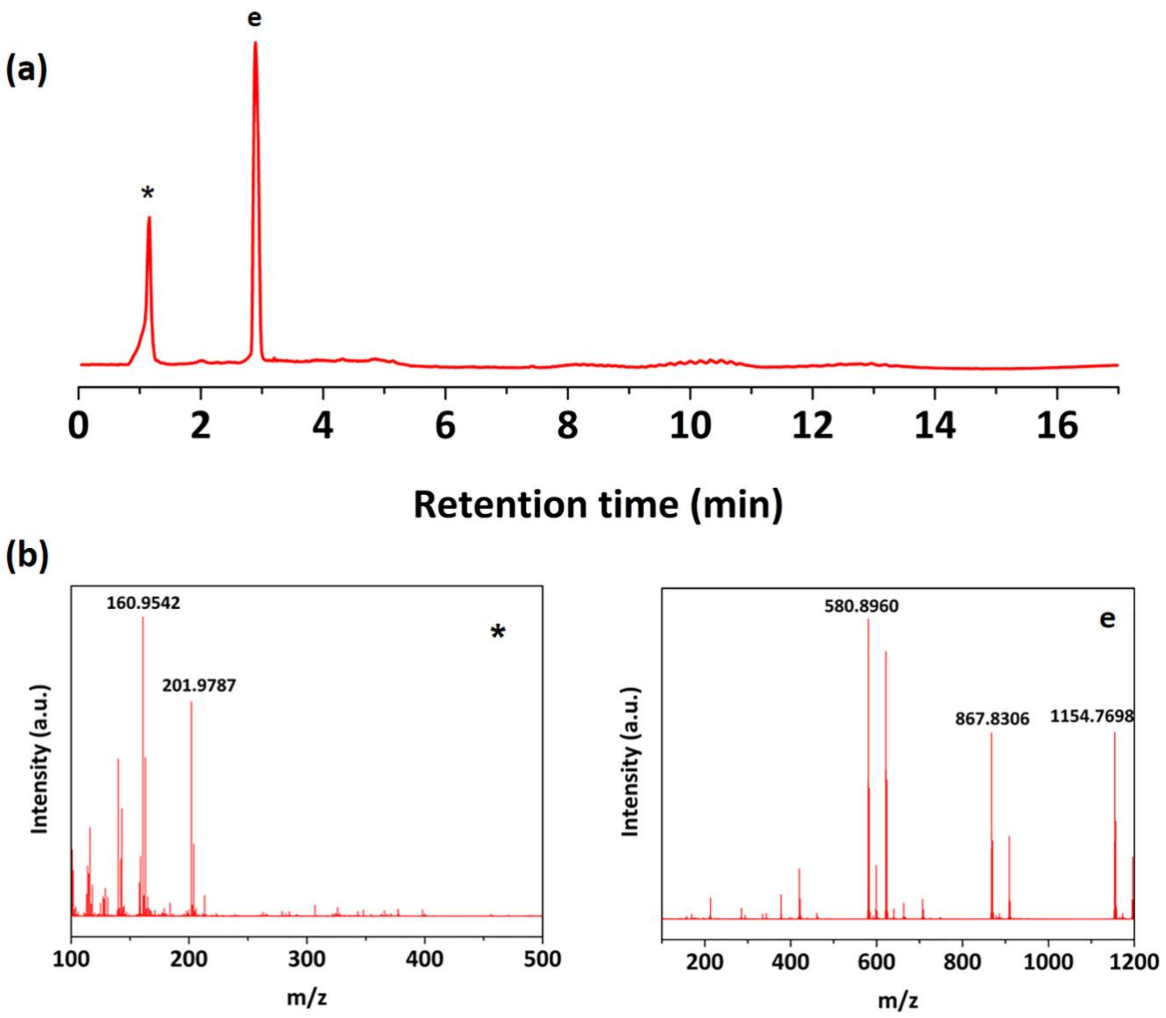

Figure S2. LC and mass spectra of the blank electrolyte.

Table S1. The proportion of each polysulfide component in the prepared catholyte and recharged electrode.

	A	B	C	D	E	F	G
**prepared**	6.7	5.8	6.4	4.2	3.4	2.0	1
**recharged**	23.3	17.9	17.3	7.3	4.1	1	-

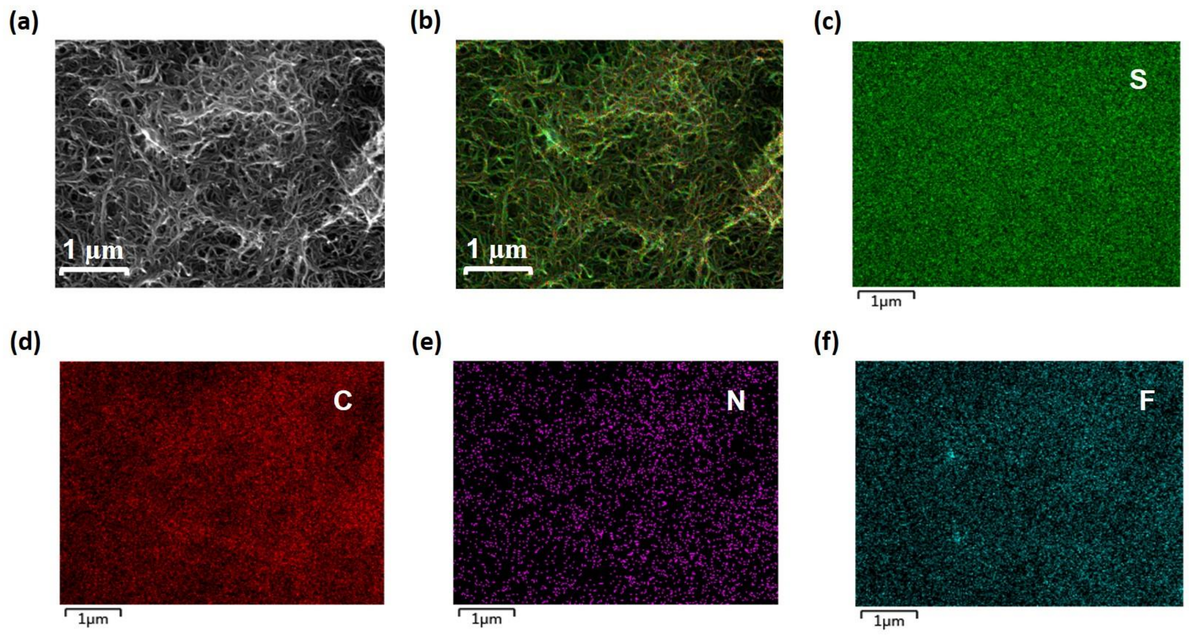

Figure S3. SEM images of the prepared cathode along with EDS elemental maps.

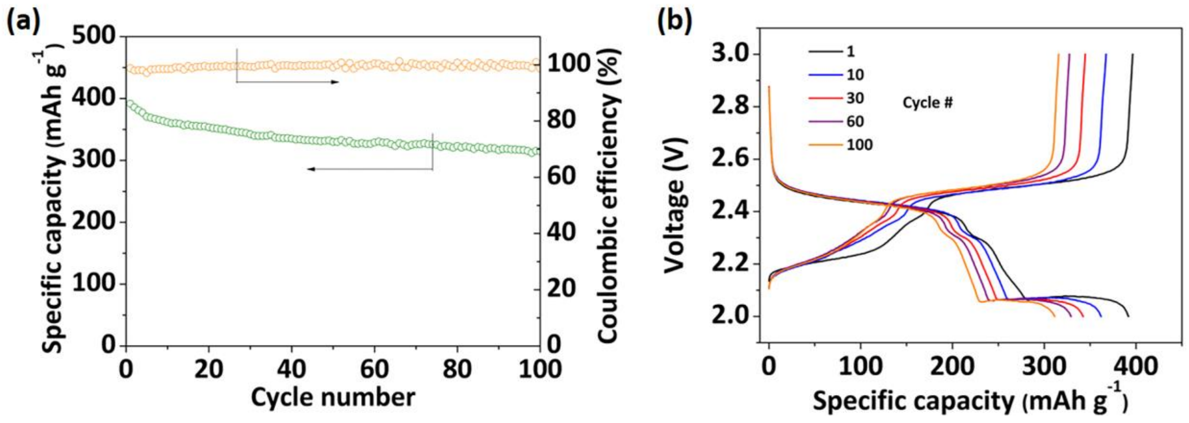

Figure S4. The cycling performance and selected voltage profiles of the Li/Py_2S_x cell at C/10 rate.

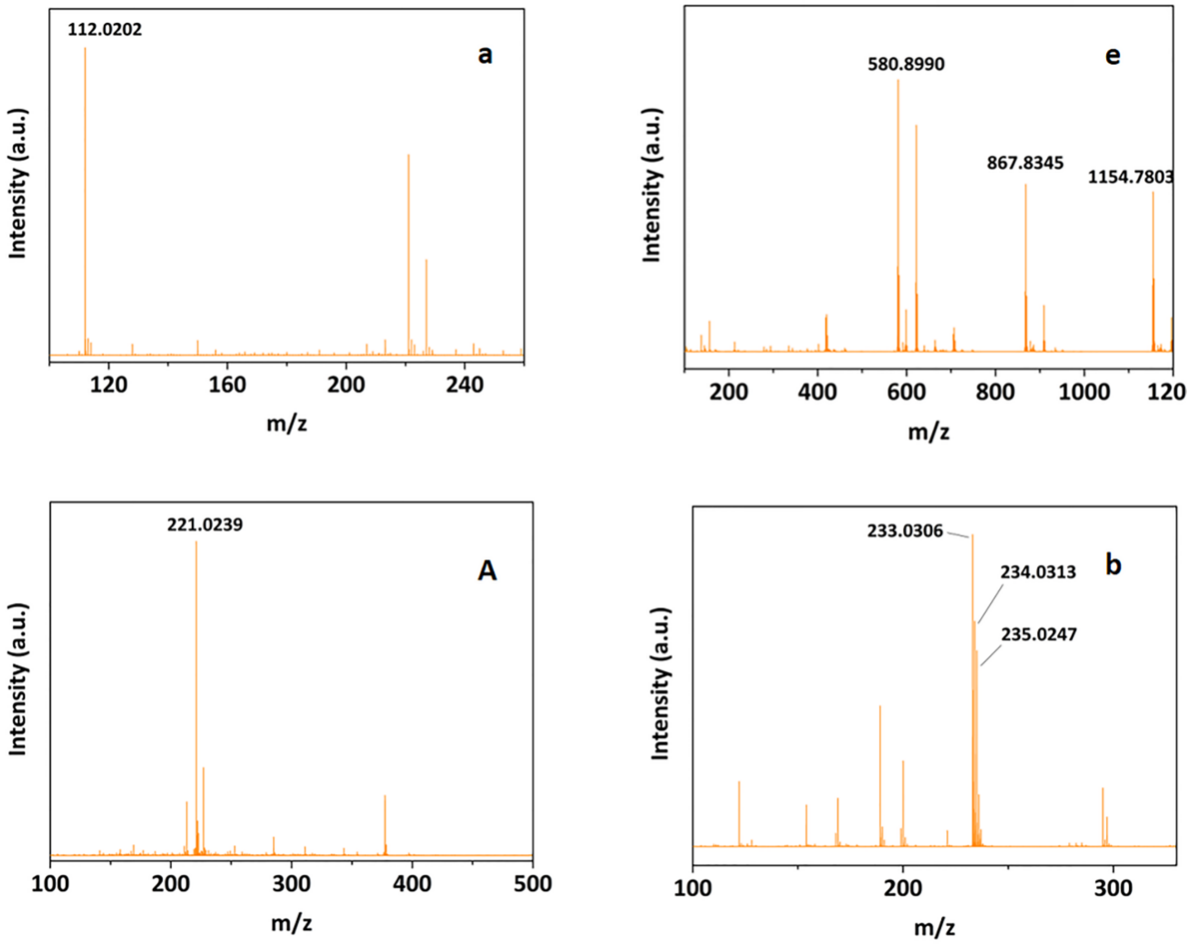

Figure S5. The mass spectra of peaks shown in the UPLC-QTof-MS spectrum (Figure 1c) of the discharged electrode.

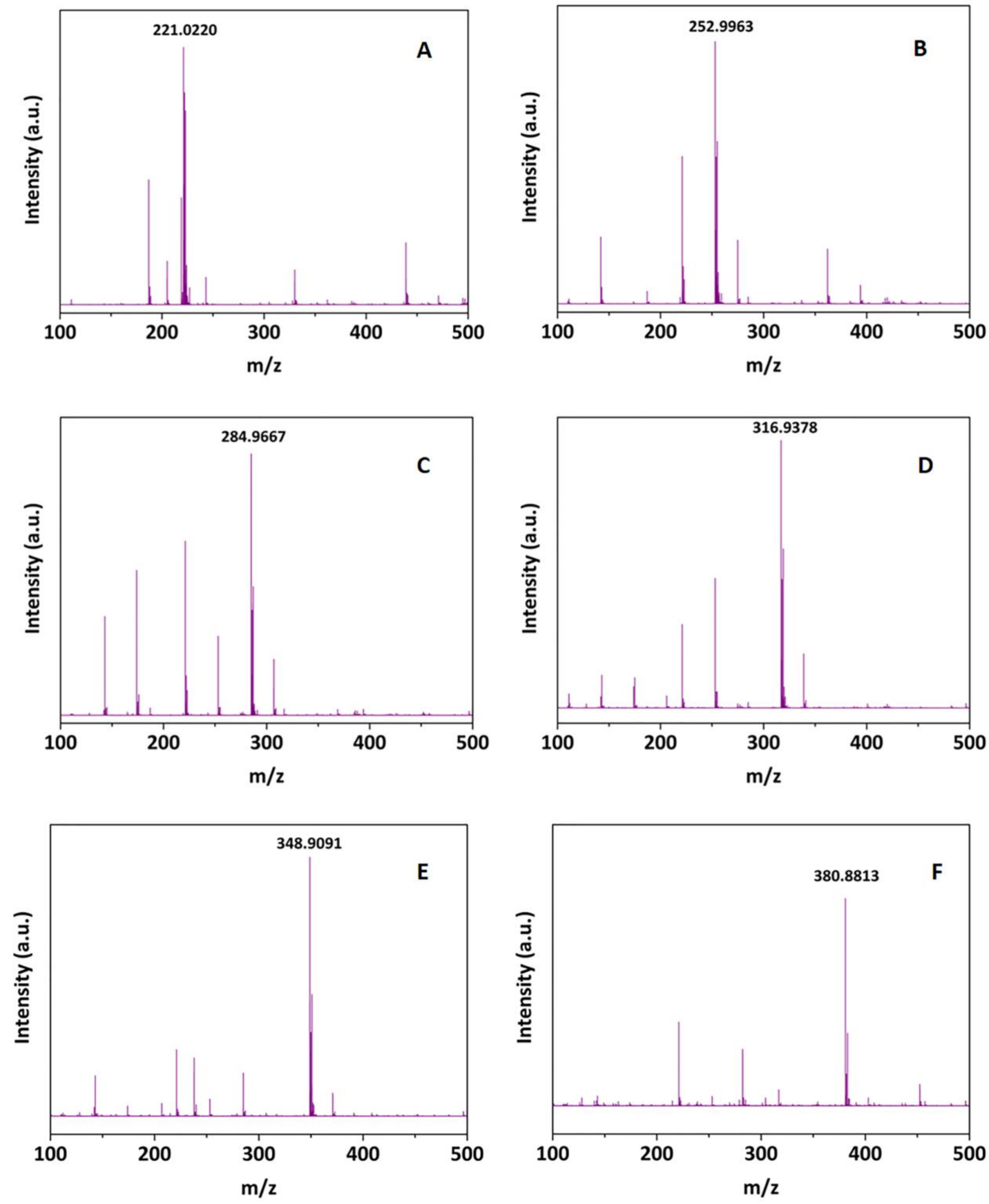

Figure S6. The mass spectra of peaks shown in the UPLC-QTof-MS spectrum (Figure 1d) of the recharged electrode.

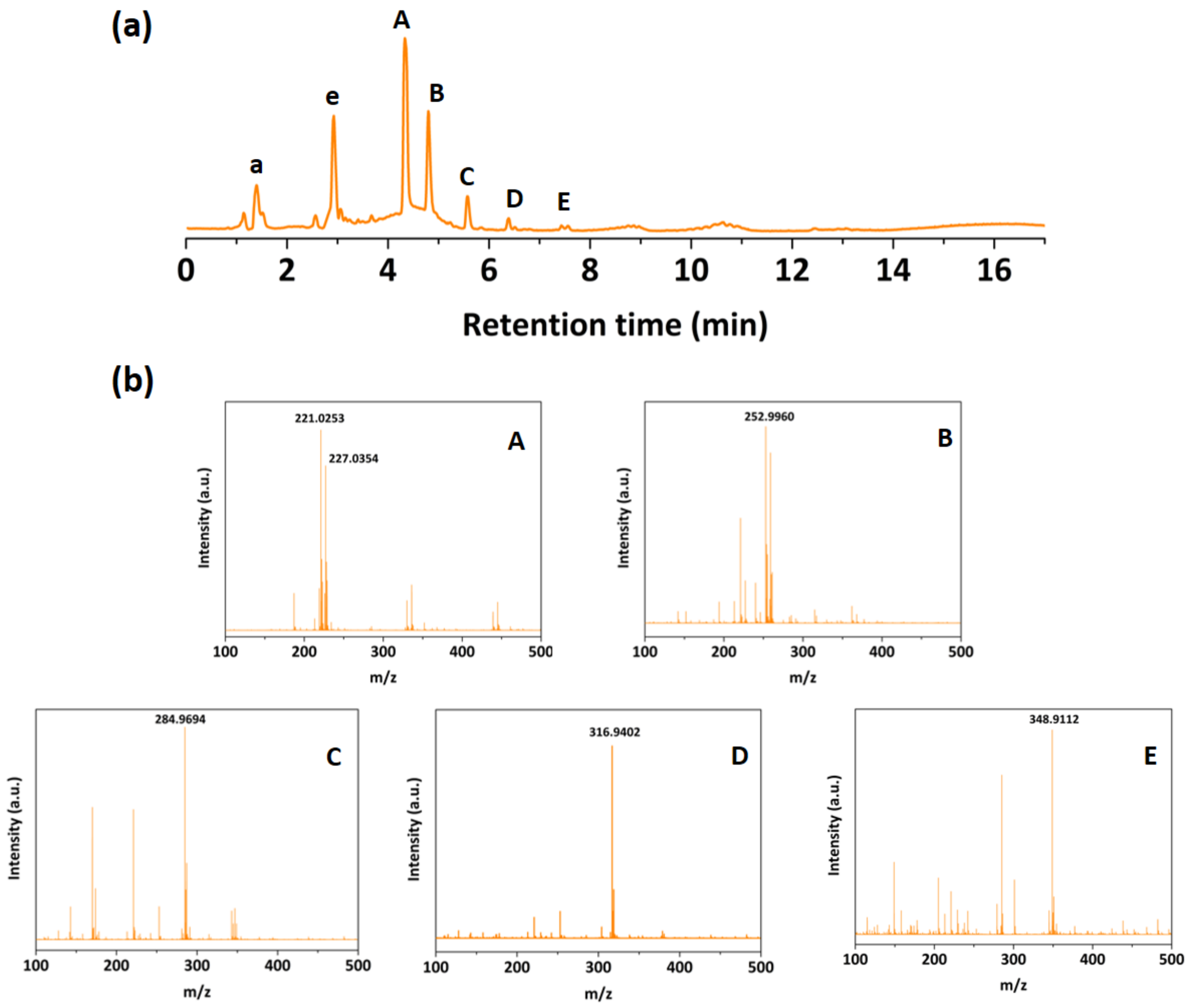

Figure S7. (a) TIC of the charged electrode after 100th cycle; (b) the mass spectra of corresponding peaks in a).

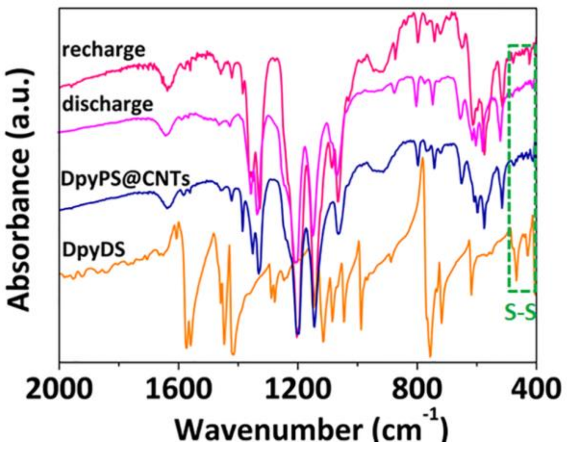

Figure S8. FTIR spectra of Py_2S_2 and Py_2S_x/CNT before and after discharge/recharge, the dotted rectangular region shows the band change due to breakage and reformation of S-S bonds.

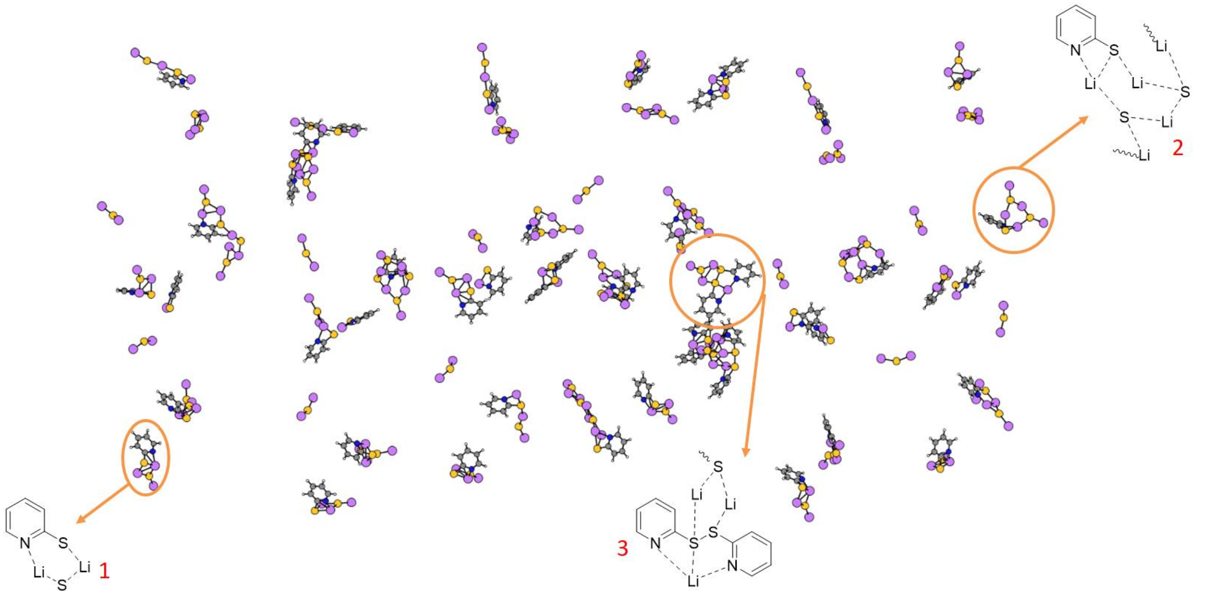

Figure S9. The partial magnified image of simulations with 792 atoms for PySLi and Li.

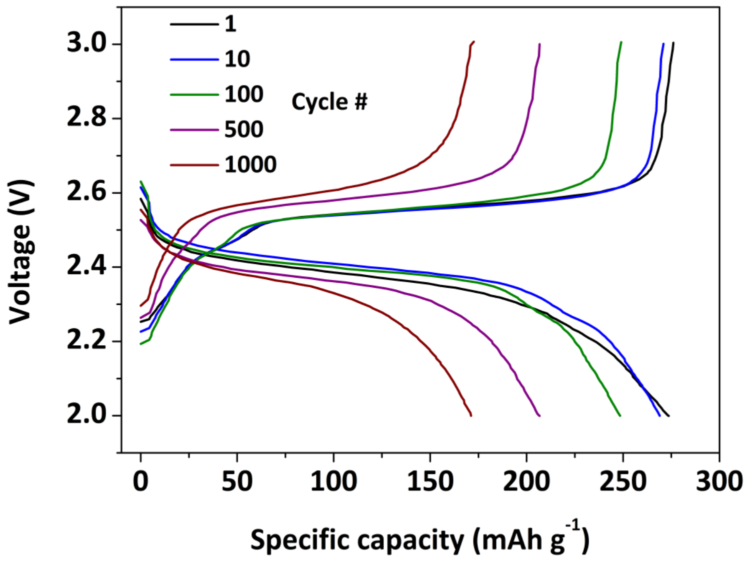

Figure S10. The selected voltage profiles of the Li/Py_2S_x cell at 5C rate.

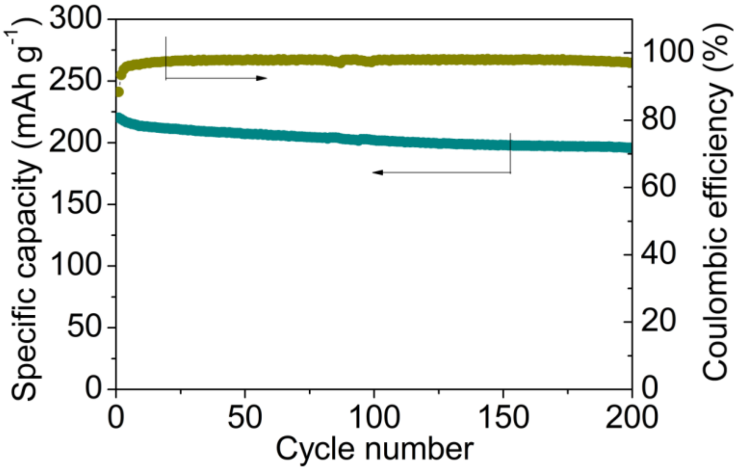

Figure S11. The cycling performacne of a Li/Py_2S_x cell with a high mass loading of 5.3 mg cm^{-2} at C/2 rate.

References:

1. Frisch, M. J.; Trucks, G. W.; Schlegel, H. B.; Scuseria, G. E.; Robb, M. A.; Cheeseman, J. R.; Scalmani, G.; Barone, B. M. V.; Petersson, G. A.; Nakatsuji, H. et al. Gaussian 09, Revision D.01, Gaussian, Inc.: Wallingford, CT, **2010**.

2. Lu, T. and Chen, F.W. Multiwfn: A multifunctional wavefunction analyzer. *J. Comput. Chem.* **2012**, *33*, 580-592.

3. Cui, Y.; Ackerson, J. D.; Ma, Y., Bhargav, A.; Karty, J. A. Guo, W.; Zhu, L. K.; Fu, Y. Z. Phenyl Selenosulfdes as Cathode Materials for Rechargeable Lithium Batteries. *Adv. Funct. Mater.* **2018**, *28*, 1801791.
